# Predictive value of peripheral blood eosinophil levels and dynamics for efficacy and safety of immune checkpoint inhibitors in non-small cell lung cancer: a real-world study

**DOI:** 10.3389/fimmu.2026.1816610

**Published:** 2026-04-30

**Authors:** Huan Zhang, Armaan Jamal, Wenrui Dou, Ge Guo, Chen Yang, Mingjun Xu, Junjie Dang, Jing Sun, Siyu Xie, Dezheng Huo, Lihua Shang

**Affiliations:** 1 Department of Medical Oncology, Harbin Medical University Cancer Hospital, Harbin, China; 2 Department of Public Health Sciences, University of Chicago, Chicago, IL, United States; 3 Cancer Prevention and Control Program, UChicago Medicine Comprehensive Cancer Center, Chicago, IL, United States; 4 Department of Clinical Medicine, Harbin Medical University, Harbin, China; 5 Department of Medical Oncology, Peking University Cancer Hospital (Inner Mongolia Campus), Affiliated Cancer Hospital of Inner Mongolia Medical University, Hohhot, China; 6 Section of Hematology and Oncology, Department of Medicine, University of Chicago, Chicago, IL, United States

**Keywords:** biomarkers, eosinophils, immunotherapy, NSCLC, retrospective cohort

## Abstract

Heterogeneous benefits from immunotherapy in advanced driver gene–negative non-small cell lung cancer (NSCLC) underscore the need for biomarkers predicting efficacy and immune-related adverse event (irAE) risk. Eosinophils, routinely measured in peripheral blood and involved in antitumor immunity, may serve as practical biomarkers for predicting the efficacy and safety of immune checkpoint inhibitors (ICIs). This retrospective study enrolled 316 consecutive patients with unresectable stage III–IV NSCLC treated with ICI-based regimens at Harbin Medical University Cancer Hospital (2016–2024). Peripheral absolute eosinophil count (AEC) and eosinophil percent (E%) were measured at baseline and after cycles 2 and 4. Overall survival (OS) and progression-free survival (PFS) were the primary and secondary endpoints. Associations with irAEs were also evaluated. Multivariable Cox analysis showed that increases in AEC and E% from baseline to cycle 4 (ΔAEC and ΔE%) were independently associated with OS (ΔAEC: aHR=0.88 per doubling, p=0.036; ΔE%: aHR=0.84 per doubling, p=0.006). Optimal cut-offs for cycle 4 AEC (0.22×10^9^/L), E% (2.60%), ΔAEC (0.01×10^9^/L), and ΔE% (0.85%), determined by the minimum p-value method, indicated that values above these thresholds were significantly associated with improved OS and PFS (p<0.05). Regarding safety, 38.4% of patients developed irAEs. Higher AEC levels at cycles 2 and 4, as well as early increases from baseline to cycle 2, were associated with an elevated risk of irAEs, while baseline AEC/E% showed no significant association with irAEs. This study supports the use of peripheral blood eosinophil-related indices as prognostic and safety biomarkers in NSCLC patients treated with ICIs.

## 1 Introduction

Immune checkpoint inhibitors (ICIs) have substantially reshaped the treatment landscape for advanced driver gene–negative non-small cell lung cancer (NSCLC), providing more durable clinical benefits than conventional chemotherapy (1–3). However, their efficacy varies substantially among individuals, and a subset of patients develop severe immune-related adverse events (irAEs) during treatment, likely driven by excessive immune activation whereby tumor-reactive T cells also attack normal tissues, including the skin, lungs, gastrointestinal tract, thyroid, pituitary gland, liver, kidneys, myocardium, and nervous system (4–6). Against this background, accurately predicting both the therapeutic benefit of ICIs and the risk of irAEs has become a crucial issue in clinical practice (7–9). To this end, various biomarkers have been proposed, including tumor PD–L1 expression, tumor mutational burden, microsatellite instability, gene expression signatures, and circulating tumor DNA (ctDNA)–based assays (10–13). Although some of these markers are now used in clinical decision-making, they all have significant limitations: most require adequate tumor tissue, rely on complex and costly platforms, are vulnerable to spatial and temporal heterogeneity, and are not routinely available in many real-world settings. Even for PD–L1, the most widely used biomarker, assessment depends on immunohistochemistry, and results can be influenced by the sampling site and specimen type, potentially failing to fully capture intratumoral heterogeneity (10–13).

By contrast, hematologic tests offer several advantages: samples are easily obtained, testing is inexpensive, and measurements can be repeated for dynamic monitoring throughout the treatment course, making them widely applicable across nearly all medical institutions. In recent years, several studies have explored the relationships between peripheral neutrophil and lymphocyte counts, their derived ratios (such as the neutrophil-to-lymphocyte ratio and platelet-to-lymphocyte ratio), and absolute eosinophil count (AEC), as well as the efficacy and toxicity of ICIs (14–18). As a routine hematologic parameter, AEC, derived from the complete blood count, is regularly incorporated into both baseline and on-treatment assessments during cancer immunotherapy. In addition to their classical roles in allergic reactions and defense against parasitic infections (19–22), eosinophils can remodel the tumor microenvironment, promote vascular normalization, and exert direct cytotoxic effects on tumor cells, thereby enhancing the antitumor activity of ICIs (23–27). Thus, eosinophilia during ICI therapy may indicate both enhanced antitumor immunity and an increased risk of irAEs (28, 29). However, conflicting evidence suggests that eosinophils may exert pro-tumor effects. Previous studies have shown that eosinophils can release multiple growth factors, such as VEGF and TGF-β, as well as matrix metalloproteinases, thereby promoting tumor angiogenesis, matrix remodeling, and the formation of an immunosuppressive microenvironment, ultimately exerting pro-tumor effects in certain contexts (30). These findings suggest that eosinophils may exhibit a dual role—both pro-tumorigenic and antitumorigenic—under varying conditions, and their true significance in cancer immunotherapy remains a matter of debate.

Recently, interest has grown in the prognostic relevance of eosinophils across multiple cancers. Evidence indicates that higher AEC in blood or tumor tissue is associated with superior responses to immunotherapy in certain entities, such as gastrointestinal cancers, head and neck cancers, melanoma, and NSCLC (31–37). By contrast, findings in Hodgkin lymphoma have been inconsistent, with some reports even suggesting worse outcomes (38). Collectively, these findings indicate that eosinophils are intimately involved in tumor immunity, yet they appear to play distinct roles in different tumor types. Despite these advances, the specific significance of eosinophils in NSCLC patients treated with ICIs remains incompletely defined. Existing NSCLC studies have primarily focused on baseline AEC, are limited by relatively small sample sizes, and have rarely incorporated eosinophil percent (E%) or on-treatment dynamic changes (28, 39–41). Moreover, most data come from ICI monotherapy cohorts, whereas evidence for ICI-based chemoimmunotherapy regimens is relatively scarce. Against this background, we conducted a retrospective analysis of NSCLC patients treated with various ICI-based regimens, systematically examining the associations of AEC, E%, and their relative changes with clinical outcomes, including OS, PFS, and the occurrence of irAEs, to further evaluate their potential predictive and prognostic value.

## 2 Study population and methods

### 2.1 Definition of the study population

**Figure 1:**
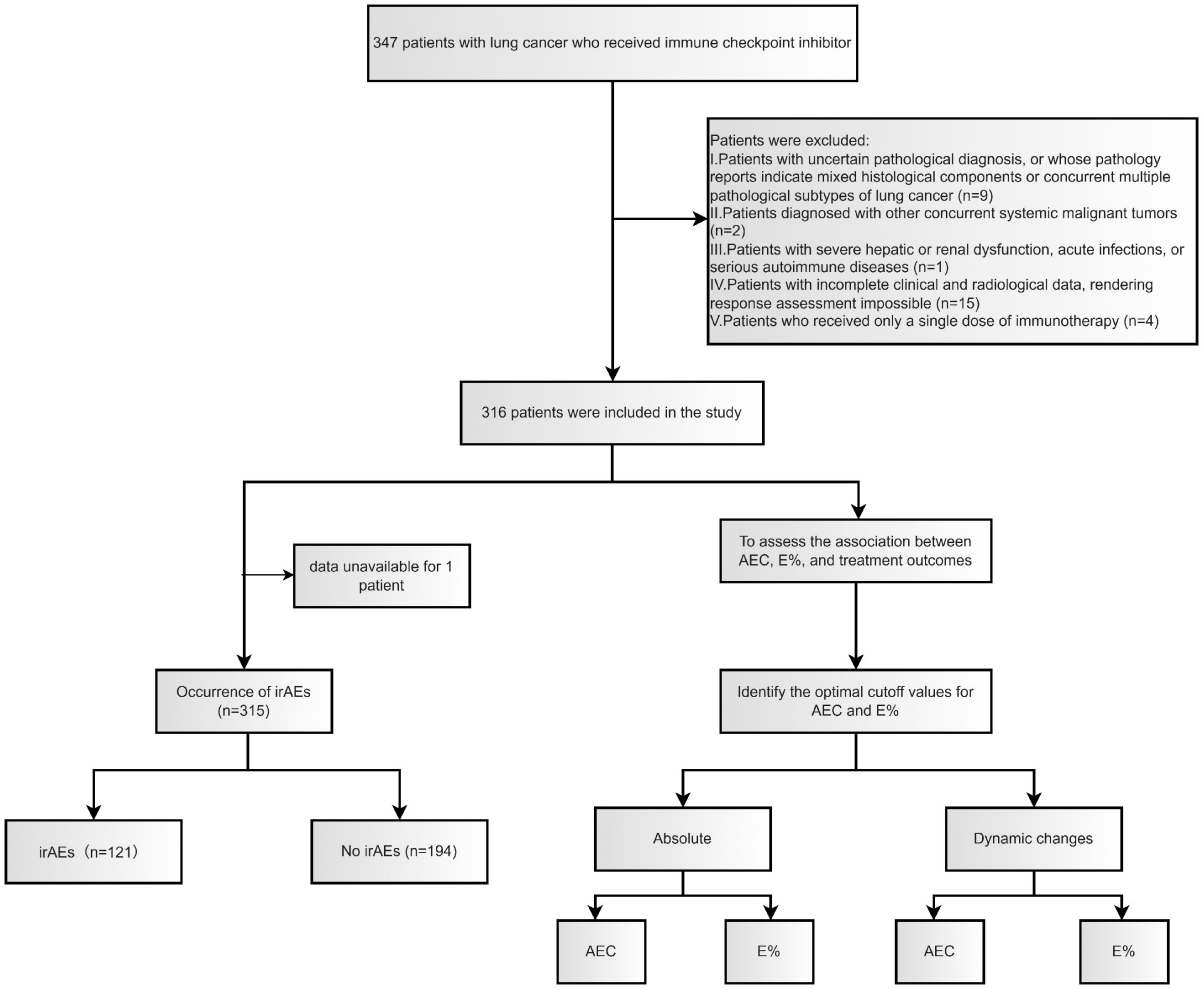
Patient selection and grouping process.

We retrospectively identified a real-world cohort of 316 patients with unresectable locally advanced or metastatic NSCLC who received ICI-based therapy at Harbin Medical University Cancer Hospital (Harbin, China) between December 2016 and December 2024. These patients were included in this retrospective study, and the overall screening and inclusion process is summarized in **Figure 1**. All patients were staged according to the 8th edition TNM classification and were treated with ICI monotherapy or ICIs combined with chemotherapy and/or anti-angiogenic agents. Follow-up continued until May 1, 2025, death, or loss to follow-up, whichever occurred first. Patients meeting all prespecified eligibility criteria were included in the final analysis. Patients were enrolled according to the following inclusion and exclusion criteria.

### 2.2 Inclusion and exclusion criteria

The following criteria were used for patient inclusion:

- Age ≥18 years
- Histologically or cytologically confirmed NSCLC
- Unresectable stage III–IV disease according to the 8th edition TNM classification
- At least one measurable lesion suitable for target-lesion selection
- Receipt of at least two cycles of ICI-based therapy
- Hematologic testing, including eosinophil measurements, performed within 7 days before initiation of immunotherapy
- Availability of complete medical records at the time of first immunotherapy

The following criteria were used for patient exclusion:

- Uncertain pathological diagnosis, or pathology reports indicating mixed histologic components or concurrent multiple pathological subtypes of lung cancer (n=9)
- Diagnosis of another concurrent active systemic malignant tumor (n=2)
- Severe hepatic or renal dysfunction, acute infection, or serious autoimmune disease at baseline (n=1)
- Incomplete clinical or radiological data that precluded response or survival assessment (n=15)
- Receipt of only a single dose of immunotherapy (n=4)

### 2.3 Data collection

We collected the following data from medical records: (1) Patient characteristics, including age, sex, smoking history, and Eastern Cooperative Oncology Group performance status (ECOG PS); (2) Tumor characteristics, including histologic subtype, disease stage, metastatic sites, and PD–L1 expression level; (3) Treatment-related variables, including line of ICI-based therapy and treatment regimen; (4) Eosinophil-related indices, including AEC and E% measured at baseline and after cycle 2 and cycle 4 of treatment; (5) Immune-related adverse events; and (6) Death and progression of diseases. Tumor response was assessed according to the Response Evaluation Criteria in Solid Tumors (RECIST), version 1.1. OS was defined as the time from initiation of ICI therapy to death or last follow-up, and PFS as the time from initiation of ICI therapy to progression, death, or last follow-up.

### 2.4 Statistical analysis

Descriptive statistics were used to summarize patient characteristics overall and by the occurrence of irAEs. Categorical variables were reported as counts and percentages and compared using Pearson’s Chi-squared test. Continuous variables were summarized as means with standard deviations (SDs) and compared using t-tests. To evaluate longitudinal trends in eosinophil levels during ICI therapy, linear mixed-effects models were used to estimate changes in AEC and E% over treatment cycles after logarithmic transformation, accounting for within-patient correlation. Adjusted regression coefficients with 95%CIs were reported.

Survival analyses were performed to assess the prognostic value of eosinophil dynamics for OS and PFS using multivariable Cox proportional hazards models. Given the potential for immortal time bias, we conducted landmark analyses at 42 days and 84 days following initiation of immunotherapy. Analyses of cycle 2 eosinophil indices and cycle 4 eosinophil indices (including changes) were restricted to patients who remained event-free and under observation at the respective landmark timepoints (42 days for cycle 2 measures and 84 days for cycle 4 measures). Adjusted hazard ratios (aHRs) with 95% confidence intervals (CIs) were presented. For prognostic stratification, optimal cut-off values for AEC and E% at cycle 4, as well as their changes from baseline to cycle 4 were determined using the minimum p-value approach, with permutation-adjusted p-values to control for multiple testing. OS was used as the outcome when selecting optimal cut-offs. Kaplan–Meier curves were generated for OS and PFS with the optimal cut-offs. Associations between eosinophil indices and irAEs were examined using multivariable logistic regression models for AEC and E% at baseline, cycle 2, and cycle 4, as well as for changes between these timepoints. We also examined the association between eosinophil indices and severe irAEs (defined as grade ≥3 vs. grade <3 or no irAEs). irAE grade was determined using CTCAE version 5.0. Eosinophil indices were log_2_-transformed, so the adjusted hazard ratios and adjusted odds ratios (aORs) were reported per doubling of the corresponding index. All multivariable models were adjusted for sex, age, smoking status, body mass index (BMI), disease stage, brain metastasis, and line of initial immunotherapy; prior surgery and radiotherapy were additionally included in selected models. Given that the myelosuppressive effects of chemotherapy may alter the natural trajectories of AEC and E%, we conducted sensitivity analyses examining longitudinal changes in eosinophil indices and their associations with survival, stratified by treatment regimen (ICI alone vs. ICI plus chemotherapy). All tests were two-sided, and p<0.05 was considered statistically significant. Statistical analyses were performed using Stata 18 (StataCorp, College Station, TX) and R version 4.4.2 (R Foundation for Statistical Computing, Vienna, Austria).

## 3 Results

### 3.1 Patient baseline characteristics

This retrospective study included 316 patients with NSCLC treated with ICI-based regimens. Of these, 192 patients (60.8%) were aged ≤65 years (**Table 1**). Most patients had good performance status, with an ECOG PS of 0–1 in 285 patients (90.2%). The majority (224, 70.9%) had stage IV disease at diagnosis. Histologic subtypes included adenocarcinoma in 180 patients (57.0%), squamous cell carcinoma in 124 (39.2%), and undifferentiated carcinoma in 12 (3.8%). Bone was the most frequent metastatic site at baseline, observed in 80 patients (25.3%). ICI-based therapy was administered as first-line treatment in 218 patients (69.0%). The most frequent treatment regimen was an ICI plus chemotherapy in 237 patients (75.0%). The median follow-up time for this cohort was 19.6 months, with an interquartile range of 13.8–32.0 months.

**Table 1:** Baseline characteristics of study participants.

Characteristic	n (%)
Age	
≤65	192 (60.8%)
>65	124 (39.2%)
Gender	
Female	91 (28.8%)
Male	225 (71.2%)
BMI, mean (SD)	23.2 (3.5)
Smoking	
No	157 (49.7%)
Yes	159 (50.3%)
ECOG PS	
0-1	285 (90.2%)
≥2	30 (9.5%)
Missing	1 (0.3%)
Tumor stage	
III	92 (29.1%)
IV	224 (70.9%)
Histological type	
Adenocarcinoma	180 (57.0%)
Squamous cell carcinoma	124 (39.2%)
Undifferentiated carcinoma	12 (3.8%)
Brain metastasis	
No	275 (87.0%)
Yes	41 (13.0%)
Liver metastasis	
No	296 (93.7%)
Yes	20 (6.3%)
Bone metastasis	
No	236 (74.7%)
Yes	80 (25.3%)
Line of initial therapy	
1	218 (69.0%)
≥2	98 (31.0%)
Surgery history	
No	224 (70.9%)
Yes	92 (29.1%)
Local radiotherapy history	
No	217 (68.7%)
Yes	99 (31.3%)
Treatment regimen	
I/I+A	40 (12.7%)
I+C	237 (75.0%)
I+C+A	39 (12.3%)
PD-L1 expression level	
< 1%	20 (6.3%)
1–49%	42 (13.3%)
≥50%	33 (10.4%)
Missing	221 (69.9%)
Follow-up time (months), median (IQR)	19.6 (13.8, 32.0)

BMI, body mass index; SD, standard deviation; ECOG PS, Eastern Cooperative Oncology Group performance status; I, immunotherapy; A, Anti-angiogenic therapy; C, chemotherapy; PD-L1, programmed death-ligand 1 IQR, interquartile range.

### 3.2 Distribution of AEC and E% over time

**Figure 2:**
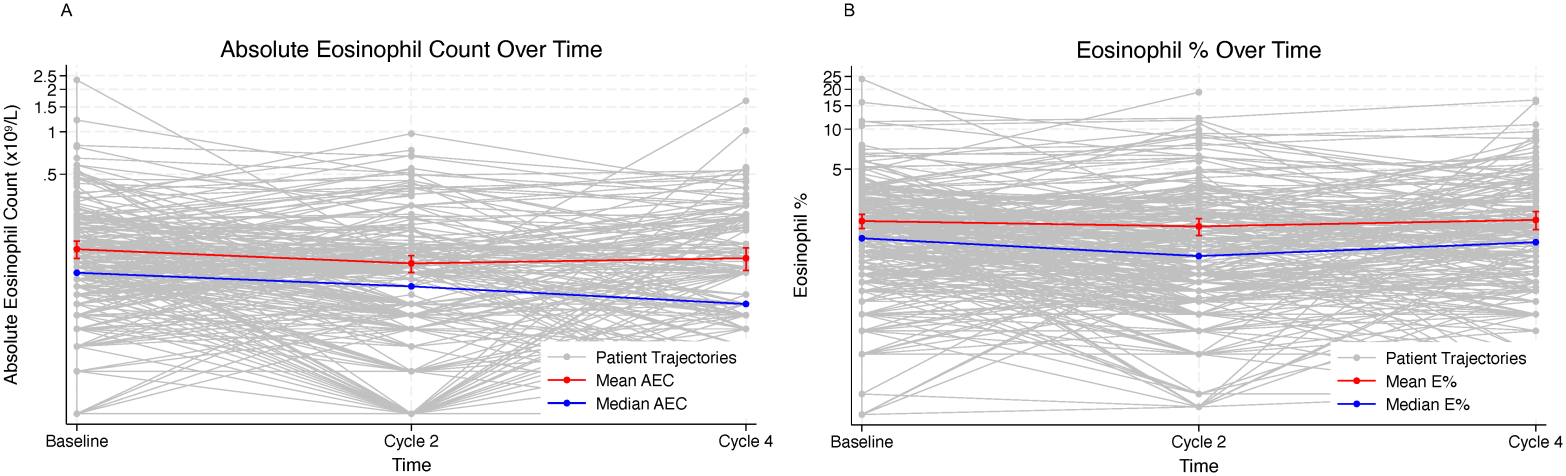
Changes in peripheral blood eosinophil-related indices from baseline to cycle 4 of immunotherapy. **(A)** Changes in the mean and median AEC from baseline to cycle 4; **(B)** Changes in the mean and median E% from baseline to cycle 4. Gray lines represent individual patients, while red and blue lines indicate the mean and median values, respectively.

AEC declined from baseline to cycle 2 and cycle 4 of ICI therapy (median 0.10→0.08→0.06×10^9^/L) (**Figure 2**, **Table 2**). E% was broadly stable with a shallow dip at cycle 2 (median 1.5%→1.1%→1.4%). In the linear mixed model adjusting for multiple demographic and clinical factors, we found, on average, AEC decreased relatively by 31% (95% CI: 22%–41%; p<0.001) at cycle 2 and by 28% (95% CI: 18%–39%; p<0.001) at cycle 4 compared to baseline (**Table 2**). In a similar manner, E% decreased relatively by 19% (95%CI: 9%–30%; p=0.001) at cycle 2 compared to baseline, while at cycle 4, there was a nonsignificant decrease of 12% (95%CI: 0–25%; p=0.069) compared to baseline.

**Table 2:** Longitudinal changes in eosinophil indices during ICI therapy and their associations with treatment cycles.

	Mean	Median	Relative fold change ^a^ (95% CI)	*P*-value
AEC, ×10^9^/L				
Baseline	0.147	0.1	1 (Reference)	
Cycle 2	0.116	0.08	0.69 (0.59, 0.78)	<0.001
Cycle 4	0.127	0.06	0.72 (0.61, 0.82)	<0.001
E%				
Baseline	2.023	1.5	1 (Reference)	
Cycle 2	1.843	1.1	0.81 (0.70, 0.91)	0.001
Cycle 4	2.062	1.4	0.88 (0.75, 1.00)	0.069

a Relative fold change estimated from linear mixed effect models, adjusted for gender, age, smoking status, body mass index, stage, brain metastasis, and line of initial therapy

AEC, absolute eosinophil count; E%, eosinophil percent; CI, confidence interval

**Supplementary Table 1** presents longitudinal changes in eosinophil indices during ICI therapy, stratified by treatment regimen (ICI alone vs. ICI plus chemotherapy). Longitudinal changes in eosinophil indices were broadly similar across the two regimen groups, though some differences emerged. AEC decreased significantly at cycle 2 in the ICI plus chemotherapy group compared to baseline, but not in the ICI alone group (interaction p=0.019), while E% showed a similar but non-significant pattern (interaction p=0.268) at cycle 2. At cycle 4, E% decreased only in the ICI alone group (interaction p=0.060).

### 3.3 Prognostic impact of AEC and E% at on-treatment timepoints

In this cohort, 147 patients died, and 241 patients had progressive diseases. AEC measured at cycle 4 was associated with improved OS, a 17% reduction in risk of death for every two-fold increase in AEC (aHR=0.83 per doubling of AEC; 95% CI: 0.73–0.94; p=0.003) in the multivariable Cox models (**Table 3**). A higher AEC at cycle 4 was also associated with improved PFS (aHR=0.91; 95% CI: 0.83–0.99; p=0.045). Eosinophil percent at cycle 4 was significantly associated with improved OS (aHR=0.81; 95% CI: 0.72–0.91; p=0.001) and PFS (aHR=0.88; 95% CI: 0.80–0.96; p=0.005) (**Table 3**). However, baseline levels and cycle 2 measurements of AEC and E% showed no significant association with either OS or PFS in the adjusted models.

**Table 3:** Association of eosinophil count and percentage at baseline, cycle 2, and cycle 4 with OS and PFS.

		Adjusted HR ^a^ (95% CI)	*P*-value
OS^b^	**Absolute Eosinophil Count**		
	Baseline	0.96 (0.86, 1.08)	0.498
	Cycle 2	1.01 (0.91, 1.13)	0.810
	Cycle 4	0.83 (0.73, 0.94)	0.003
	**Eosinophil Percent**		
	Baseline	0.94 (0.85, 1.04)	0.237
	Cycle 2	0.92 (0.83, 1.03)	0.158
	Cycle 4	0.81 (0.72, 0.91)	0.001
PFS^b^	**Absolute Eosinophil Count**		
	Baseline	0.99 (0.91, 1.08)	0.854
	Cycle 2	1.05 (0.96, 1.14)	0.293
	Cycle 4	0.91 (0.83, 0.99)	0.045
	**Eosinophil Percent**		
	Baseline	0.98 (0.90, 1.06)	0.596
	Cycle 2	0.96 (0.88, 1.05)	0.421
	Cycle 4	0.88 (0.80, 0.96)	0.005

a Adjusted hazard ratio per doubling of eosinophil indices in Cox proportional hazard models, adjusted for gender, age, smoking, body mass index, stage, brain metastasis, and line of initial therapy.

b For cycle 2 and cycle 4 analyses, landmark times were set at 42 and 84 days after initiation of immune checkpoint inhibitor therapy, respectively.

HR, hazard ratio; CI, confidence interval; OS, overall survival; PFS, progression-free survival A p-value < 0.05 indicates statistical significance in this study.

### 3.4 Changes in AEC and E% during treatment and associations with survival outcomes

Dynamic changes in peripheral eosinophil levels during ICI therapy were further examined to investigate their relationship with survival outcomes. For OS (**Table 4**), doubling in AEC from baseline to cycle 4 (aHR=0.88; 95%CI: 0.78–0.99; p=0.036) and from cycle 2 to cycle 4 (aHR=0.88; 95%CI: 0.78–0.99; p=0.028) were significantly associated with improved OS. Consistently, doubling in E% from baseline to cycle 4 was also associated with improved OS (aHR=0.84, 95%CI: 0.73–0.95, p=0.006), and doubling in E% from cycle 2 to cycle 4 showed a borderline trend toward improved OS (aHR=0.89, 95%CI: 0.79–1.01, p=0.062). Doubling AEC from cycle 2 to cycle 4 was significantly associated with longer PFS (aHR=0.90, 95% CI: 0.82–0.99, p=0.025). Similarly, doubling in E% from baseline to cycle 4 was also associated with longer PFS (aHR=0.91, 95% CI: 0.83–0.99, p=0.035), and doubling in E% from cycle 2 to cycle 4 showed a borderline trend toward longer PFS (aHR=0.92, 95% CI: 0.84–1.01, p=0.092).

**Table 4:** Association of changes in eosinophil count and percentage with OS and PFS.

		Adjusted HR ^a^ (95% CI)	*P*-value
**OS** ^b^	**Absolute Eosinophil Count**		
	Change Baseline to Cycle 2	1.03 (0.91, 1.15)	0.655
	Change Baseline to Cycle 4	0.88 (0.78, 0.99)	0.036
	Change Cycle 2 to Cycle 4	0.88 (0.78, 0.99)	0.028
	**Eosinophil Percent**		
	Change Baseline to Cycle 2	0.94 (0.83, 1.06)	0.315
	Change Baseline to Cycle 4	0.84 (0.73, 0.95)	0.006
	Change Cycle 2 to Cycle 4	0.89 (0.79, 1.01)	0.062
**PFS** ^b^	**Absolute Eosinophil Count**		
	Change Baseline to Cycle 2	1.03 (0.94, 1.13)	0.491
	Change Baseline to Cycle 4	0.93 (0.85, 1.02)	0.126
	Change Cycle 2 to Cycle 4	0.90 (0.82, 0.99)	0.025
	**Eosinophil Percent**		
	Change Baseline to Cycle 2	0.95 (0.87, 1.04)	0.290
	Change Baseline to Cycle 4	0.91 (0.83, 0.99)	0.035
	Change Cycle 2 to Cycle 4	0.92 (0.84, 1.01)	0.092

a Adjusted hazard ratio per doubling of eosinophil indices in Cox proportional hazard models, adjusted for gender, age, smoking, body mass index, stage, brain metastasis, and line of initial therapy.

b For cycle 2 and cycle 4 analyses, landmark times were set at 42 and 84 days after initiation of immune checkpoint inhibitor therapy, respectively. HR, hazard ratio; CI, confidence interval; OS, overall survival; PFS, progression-free survival. A p-value < 0.05 indicates statistical significance in this study.

**Supplementary Table 2** presents associations between eosinophil indices and OS/PFS, stratified by treatment regimen. Eosinophil indices were not associated with survival outcomes in the ICI alone group, whereas, in most cases, higher cycle 4 values and changes to cycle 4 were associated with improved OS and PFS in the ICI plus chemotherapy group. In addition, associations of eosinophil indices with OS/PFS were generally not significantly different between the two groups (p for interaction >0.05), except for E% at cycle 4 and change in E% from baseline to cycle 4 with respect to OS (interaction p=0.016 and 0.026, respectively). Specifically, higher E% at cycle 4 was associated with improved OS in the ICI plus chemotherapy group (aHR=0.77, 95% CI 0.67–0.88; p < 0.001), but not in the ICI alone group (aHR=1.17, 95% CI 0.85–1.61; p = 0.336). Similarly, increases in E% from baseline to cycle 4 were associated with improved OS in the ICI plus chemotherapy group (aHR=0.79, 95% CI 0.68–0.92; p = 0.003), but not in the ICI alone group (aHR=1.11, 95% CI 0.85–1.45; p = 0.444).

### 3.5 Cut-offs in peripheral AEC and E% for prognosis

**Figure 3:**
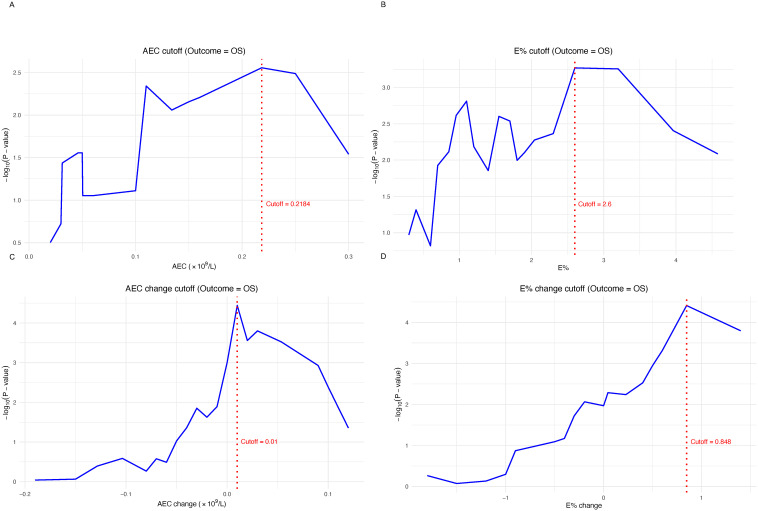
Determination of the optimal cutoff values for eosinophil-related indices using OS as the outcome. The minimum p-value approach was applied to determine the optimal cutoff values for **(A)** optimal cutoff value of AEC after cycle 4 of treatment; **(B)** optimal cutoff value of E% after cycle 4 of treatment; **(C)** optimal cutoff value of the increase in ΔAEC from baseline to after cycle 4 of treatment; **(D)** optimal cutoff value of the increase in ΔE% from baseline to after cycle 4 of treatment. Red dashed lines indicate the cutoff points corresponding to the highest −log10(p-value).

To establish clinically relevant thresholds for eosinophil-based prognostic markers, we determined optimal cut-off values for AEC and E% at cycle 4, as well as for ΔAEC and ΔE% from baseline to cycle 4. Using the minimum p-value approach with permutation adjustment, we identified the values that best stratified patients according to OS. The optimal cut-off for AEC at cycle 4 was 0.22×10^9^/L (**Figure 3A**), and the optimal cut-off for E% at cycle 4 was 2.60% (**Figure 3B**). For the increases from baseline to cycle 4, the optimal cut-offs were 0.01×10^9^/L for ΔAEC (**Figure 3C**) and 0.85% for ΔE% (**Figure 3D**). These cut-offs were used to dichotomize patients into higher versus lower groups (for cycle 4 levels) and markedly increased versus non–markedly increased groups (for ΔAEC and ΔE%).

**Figure 4:**
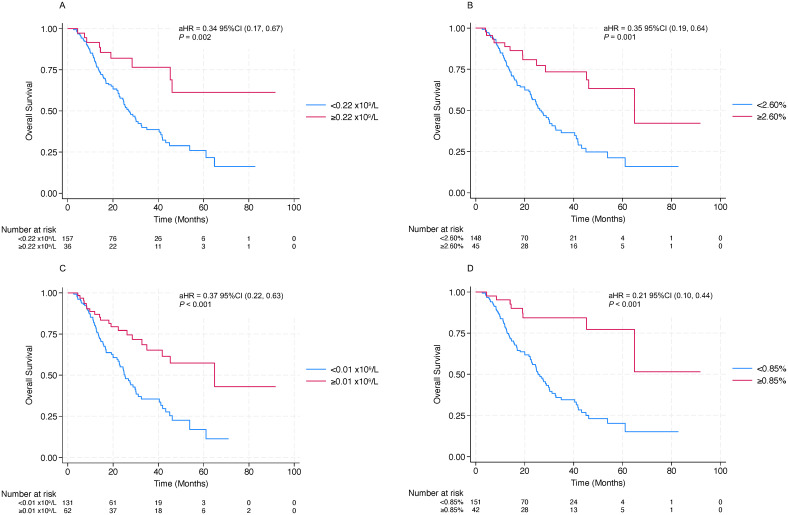
OS stratified by eosinophil indices. Kaplan–Meier curves of OS stratified by **(A)** AEC at cycle 4; **(B)** E% at cycle 4; **(C)** increases in AEC (ΔAEC) from baseline to after cycle 4 of treatment; **(D)** increases in E% (ΔE%) from baseline to after cycle 4 of treatment, according to the optimal cutoff values. .

**Figure 5:**
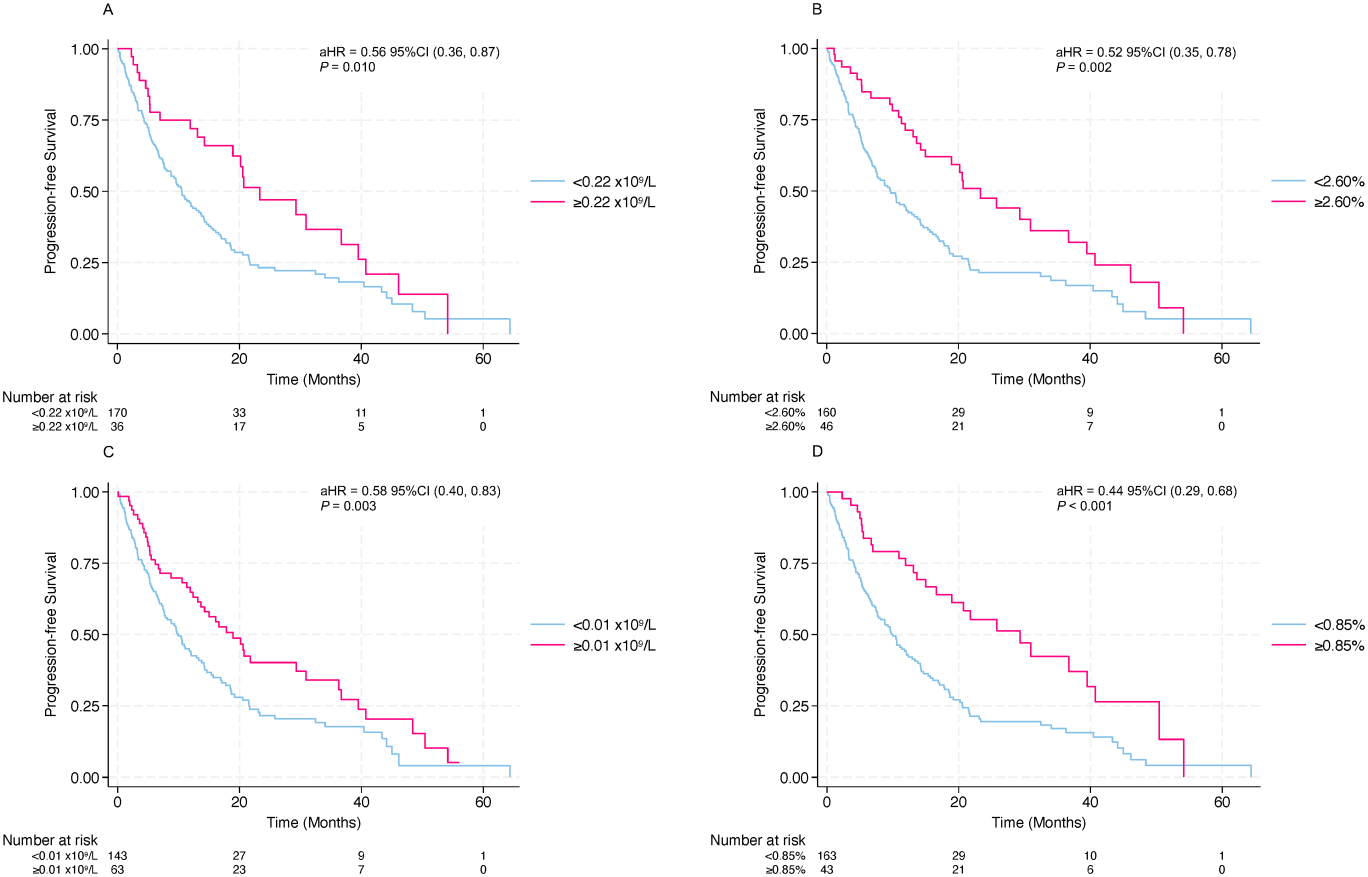
PFS stratified by eosinophil indices. Kaplan–Meier curves of PFS stratified by **(A)** AEC at cycle 4; **(B)** E% at cycle 4; **(C)** increases in AEC (ΔAEC) from baseline to after cycle 4 of treatment; **(D)** increases in E% (ΔE%) from baseline to after cycle 4 of treatment, according to the optimal cutoff values.

Patients were grouped according to the cut-off values defined above. For AEC, patients in the higher cycle 4 AEC group (≥0.22×10^9^/L) showed better OS (aHR=0.34; p=0.002; **Figure 4A**) and likewise had an advantage in PFS (aHR=0.56; p=0.010; **Figure 5A**). For E%, patients in the higher cycle 4 E% group (≥2.60%) showed clearly better OS curve (aHR=0.35; p=0.001; **Figure 4B**), with concordant improvement in PFS (aHR=0.52; p=0.002; **Figure 5B**). When examining increases from baseline to the cycle 4 timepoint, patients in the increased ΔAEC group (ΔAEC≥0.01×10^9^/L) experienced significantly better OS (aHR=0.37; p<0.001; **Figure 4C**) and likewise showed improved PFS (aHR=0.58; p=0.003; **Figure 5C**). Similarly, patients in the markedly increased ΔE% group (≥0.85%) had significantly longer OS (aHR=0.21; p<0.001; **Figure 4D**), with a concordant better PFS (aHR=0.44; p<0.001; **Figure 5D**). Taken together, compared with their corresponding lower or non–markedly increased counterparts, patients in the higher cycle 4 AEC and E% groups, as well as those in the markedly increased ΔAEC and ΔE% groups, experienced more favorable survival outcomes under ICI therapy.

### 3.6 Relationship of AEC and E% with any irAEs

Baseline characteristics stratified by irAE occurrence are summarized in **Table 5**. Of the 316 patients, 121 developed irAEs, 194 did not, and 1 patient had on data on irAEs. Patients with and without irAEs were well-balanced with respect to age, BMI, smoking status, tumor stage, brain metastasis, treatment line, treatment regimen, and PD–L1 expression. Borderline differences were observed in sex distribution (p=0.070), histology (p=0.089), and ECOG performance status (p=0.072). Compared with patients without irAEs, the irAE group had significantly higher proportions of prior surgery and local radiotherapy (p<0.001).

**Table 5:** Participant characteristics by immune-related adverse events.

Characteristic	No IR adverse event (n=194) n (%)	IR adverse event (n=121) n (%)	*P*-value ^a^
Age			0.53
≤65	115 (59.3%)	76 (62.8%)	
>65	79 (40.7%)	45 (37.2%)	
Gender			0.07
Female	49 (25.3%)	42 (34.7%)	
Male	145 (74.7%)	79 (65.3%)	
**BMI, mean (SD)**	23.0 (3.3)	23.5 (3.7)	**0.19**
Smoking Status			0.28
No	92 (47.4%)	65 (53.7%)	
Yes	102 (52.6%)	56 (46.3%)	
ECOG PS			0.072
0-1	170 (87.6%)	114 (94.2%)	
≥2	23 (11.9%)	7 (5.8%)	
Missing	1 (0.5%)	0 (0.0%)	
Histological Type			0.089
AC	107 (55.2%)	72 (59.5%)	
SCC	76 (39.2%)	48 (39.7%)	
UC	11 (5.6%)	1 (0.8%)	
Tumor Stage			0.31
III	60 (30.9%)	31 (25.6%)	
IV	134 (69.1%)	90 (74.4%)	
Brain Metastasis			0.55
No	167 (86.1%)	107 (88.4%)	
Yes	27 (13.9%)	14 (11.6%)	
Line of Initial Therapy			0.31
1	131 (67.5%)	86 (71.1%)	
2	36 (18.6%)	25 (20.7%)	
≥3	27 (13.9%)	10 (8.3%)	
Surgery History			<0.001
No	105 (86.8%)	119 (61.3%)	
Yes	16 (13.2%)	75 (38.7%)	
Local Radiotherapy History			<0.001
No	107 (88.4%)	110 (56.7%)	
Yes	14 (11.6%)	84 (43.3%)	
Treatment Regimen			0.46
I/I+A	26 (13.4%)	14 (11.6%)	
I+C	148 (76.3%)	89 (73.6%)	
I+C+A	20 (10.3%)	18 (14.9%)	
PD-L1 Expression Level			0.21
<1%	14 (7.2%)	5 (4.1%)	
1–49%	21 (10.8%)	21 (17.4%)	
≥50%	20 (10.3%)	13 (10.7%)	
Missing	139 (71.6%)	82 (67.8%)	

a *P-*value was computed using Pearson’s Chi-squared test, or t-test, where appropriate.

IR, immune-related; BMI, body mass index; SD, standard deviation; ECOG PS, Eastern Cooperative Oncology Group performance status; AC, adenocarcinoma; SCC, squamous cell carcinoma; UC, undifferentiated carcinoma; I, immunotherapy; A, Anti-angiogenic therapy; C, chemotherapy; PD-L1, programmed death-ligand 1. A p-value < 0.05 indicates statistical significance in this study.

The associations of AEC and E% with the occurrence of any irAEs are presented in **Table 6**. Baseline AEC levels were not associated with the development of irAEs. However, higher AEC values at cycle 2 and cycle 4 were significantly associated with an increased likelihood of irAE occurrence (cycle 2: aOR=1.49 per doubling in AEC; 95%CI: 1.24–1.80; p<0.001; cycle 4: aOR=1.28; 95%CI: 1.04–1.56; p=0.019). An earlier increase in AEC better predicted irAEs (baseline to cycle 2: aOR=1.44; 95%CI: 1.19–1.75; p<0.001), whereas changes from baseline to cycle 4 and from cycle 2 to cycle 4 were not associated with irAE risk (p>0.05). In contrast, for E%, neither the absolute values nor the changes showed a significant association with irAE.

**Table 6:** Association between eosinophil count, %, or changes and any immune-related adverse events.

	Unadjusted OR (95% CI)	*P*-value	Adjusted OR ^a^ (95% CI)	*P*-value	Adjusted OR ^b^ (95% CI)	*P*-value
Absolute eosinophil count						
Baseline	1.12 (0.95, 1.31)	0.184	1.14 (0.96, 1.35)	0.126	1.09 (0.91, 1.29)	0.353
Cycle 2	1.43 (1.21, 1.69)	<0.001	1.49 (1.25, 1.77)	<0.001	1.49 (1.24, 1.80)	<0.001
Cycle 4	1.22 (1.03, 1.46)	0.022	1.22 (1.02, 1.47)	0.033	1.28 (1.04, 1.56)	0.019
Change Baseline to Cycle 2	1.39 (1.17, 1.65)	<0.001	1.41 (1.18, 1.69)	<0.001	1.44 (1.19, 1.75)	<0.001
Change Baseline to Cycle 4	1.10 (0.94, 1.29)	0.215	1.09 (0.92, 1.28)	0.337	1.13 (0.94, 1.36)	0.188
Change Cycle 2 to Cycle 4	0.91 (0.78, 1.06)	0.220	0.89 (0.75, 1.04)	0.148	0.94 (0.78, 1.13)	0.527
Eosinophil percent						
Baseline	1.13 (0.97, 1.32)	0.113	1.16 (0.98, 1.36)	0.078	1.13 (0.96, 1.34)	0.146
Cycle 2	1.11 (0.95, 1.30)	0.203	1.13 (0.96, 1.34)	0.146	1.12 (0.93, 1.33)	0.225
Cycle 4	0.97 (0.81, 1.15)	0.694	0.96 (0.80, 1.15)	0.624	1.00 (0.82, 1.22)	0.984
Change Baseline to Cycle 2	1.03 (0.88, 1.21)	0.702	1.03 (0.87, 1.22)	0.735	1.03 (0.86, 1.24)	0.715
Change Baseline to Cycle 4	0.91 (0.77, 1.07)	0.271	0.89 (0.75, 1.06)	0.203	0.92 (0.76, 1.12)	0.407
Change Cycle 2 to Cycle 4	0.92 (0.78, 1.09)	0.334	0.90 (0.76, 1.08)	0.266	0.96 (0.79, 1.17)	0.703

a Odds ratio per doubling of eosinophil indices in multivariable logistic regression adjusted for gender, age, smoking status, body mass index, stage, brain metastasis, and line of initial therapy.

b Odds ratio per doubling of eosinophil indices in multivariable logistic regression adjusted for gender, age, smoking status, body mass index, stage, brain metastasis, line of initial therapy, surgery, and radiotherapy.

OR, odds ratio; CI, confidence interval

### 3.7 Relationship of AEC and E% with severe irAEs

Of the 121 patients with an irAE, 97 had an irAE of grade <3 and 24 had an irAE of grade ≥3. Corticosteroids were given to 24 patients with grade ≥3 irAE and to one patient with grade <3 irAE. The associations of AEC and E% with the occurrence of severe irAEs are presented in **Table 7**. Higher AEC values at cycle 2 were significantly associated with the occurrence of a severe irAE (cycle 2: aOR=2.40; 95% CI: 1.52–3.79; p<0.001). An earlier increase in AEC was also associated with severe irAEs (baseline to cycle 2: aOR=1.81; 95% CI: 1.22–2.70; p=0.003). On the other hand, an increase from cycle 2 to cycle 4 showed a borderline trend towards lower odds of severe irAEs (cycle 2 to cycle 4: aOR=0.66; 95% CI: 0.43–1.02; p=0.061). In terms of E%, a similar trend was seen. Higher values at cycle 2 were significantly associated with the occurrence of a severe irAE (cycle 2: aOR=1.78; 95% CI: 1.21–2.61; p=0.003). An earlier increase showed a borderline trend towards higher odds of severe irAEs (baseline to cycle 2: aOR=1.44; 95% CI: 0.99–2.10; p=0.054). Conversely, an increase from cycle 2 to cycle 4 was associated with lower odds of severe irAEs (cycle 2 to cycle 4: aOR=0.65; 95% CI: 0.42–0.99; p=0.046). We also observed that AEC at cycles 2 and 4, and E% at baseline and cycle 2, were elevated with increasing irAE severity (**Supplementary Table 3**).

**Table 7:** Association between eosinophil count, %, or changes and severe immune-related adverse events^a^.

	Unadjusted OR (95% CI)	*P*-value	Adjusted OR ^b^ (95% CI)	*P*-value	Adjusted OR ^c^ (95% CI)	*P-*value
Absolute eosinophil count						
Baseline	1.28 (0.94, 1.74)	0.118	1.22 (0.89, 1.67)	0.209	1.16 (0.86, 1.58)	0.337
Cycle 2	2.39 (1.56, 3.66)	<0.001	2.39 (1.53, 3.73)	<0.001	2.40 (1.52, 3.79)	<0.001
Cycle 4	1.18 (0.82, 1.70)	0.369	1.14 (0.77, 1.70)	0.505	1.11 (0.74, 1.68)	0.602
Change Baseline to Cycle 2	1.69 (1.18, 2.43)	0.004	1.81 (1.22, 2.69)	0.003	1.81 (1.22, 2.70)	0.003
Change Baseline to Cycle 4	0.92 (0.66, 1.28)	0.626	0.92 (0.65, 1.32)	0.654	0.91 (0.63, 1.30)	0.593
Change Cycle 2 to Cycle 4	0.67 (0.47, 0.96)	0.030	0.65 (0.43, 0.99)	0.043	0.66 (0.43, 1.02)	0.061
Eosinophil percent						
Baseline	1.17 (0.87, 1.57)	0.302	1.13 (0.84, 1.52)	0.416	1.09 (0.82, 1.46)	0.552
Cycle 2	1.84 (1.27, 2.66)	0.001	1.80 (1.23, 2.65)	0.003	1.78 (1.21, 2.62)	0.003
Cycle 4	0.94 (0.66, 1.35)	0.754	0.90 (0.61, 1.33)	0.583	0.90 (0.60, 1.35)	0.611
Change Baseline to Cycle 2	1.37 (0.98, 1.91)	0.062	1.41 (0.98, 2.03)	0.065	1.44 (0.99, 2.10)	0.054
Change Baseline to Cycle 4	0.79 (0.56, 1.11)	0.177	0.77 (0.52, 1.15)	0.201	0.77 (0.52, 1.16)	0.215
Change Cycle 2 to Cycle 4	0.65 (0.46, 0.92)	0.016	0.65 (0.43, 0.97)	0.037	0.65 (0.42, 0.99)	0.046

a Severe irAEs was categorized as grade ≥3 versus grade <3 or no irAE.

b Odds ratio per doubling of eosinophil indices in multivariable logistic regression adjusted for gender, age, smoking status, body mass index, stage, and brain metastasis, and line of initial therapy.

c Odds ratio per doubling of eosinophil indices in multivariable logistic regression adjusted for gender, age, smoking status, body mass index, stage, brain metastasis, line of initial therapy, surgery, and radiotherapy.

OR, odds ratio; CI, confidence interval

## 4 Discussion

In this study, we evaluated the associations of AEC and E% with treatment outcomes in a real-world cohort of NSCLC patients receiving ICIs. We found that higher AEC and E% at cycle 4 were associated with improved OS, whereas E% at cycle 4 significantly associated with longer PFS. We also suggested optimal cut-off values of AEC and E% at cycle 4 for predicting OS, which may be validated in future studies. In contrast, baseline AEC and baseline E% showed no statistically significant associations with OS or PFS in this study. In previous studies, Takeuchi et al. reported that patients with an intermediate baseline eosinophil count (0.1–0.5×10^9^/L) had a longer median OS, whereas those with baseline AEC≥0.5×10^9^/L or <0.1×10^9^/L had shorter median OS (40). Similarly, Chu et al. found that higher baseline AEC was associated with longer PFS (42). Sibille et al. reported that baseline eosinophil indices were not significantly associated with objective response or disease control. However, early on-treatment increases in relative eosinophil counts at the first and second radiological evaluation timepoints were correlated with higher objective response rates and longer treatment durations (43). In real-world clinical practice, baseline eosinophil indices are inherently unstable. Elevated baseline AEC may be related to allergic reactions or chronic inflammatory conditions, and many short-term factors (such as glucocorticoid use or acute infection) can markedly influence peripheral AEC at baseline (44, 45). Therefore, a higher baseline eosinophil level does not necessarily indicate ongoing effective antitumor immunity in routine practice. Our results suggest that on-treatment eosinophil indices and their dynamic changes may better reflect patients’ actual status in everyday clinical environments than baseline measures, thereby providing greater clinical utility as predictive markers.

Although eosinophils have traditionally been regarded as classical inflammatory effector cells, an increase in peripheral blood AEC or E% during ICI therapy may more likely reflect treatment-related immune activation rather than a merely nonspecific inflammatory response. Current evidence suggests that eosinophils can be recruited and activated under the regulation of cytokines such as IL-5, IL-33, and GM-CSF (46), and may enhance antitumor immunity by secreting chemokines including CCL5, CXCL9, and CXCL10, thereby promoting the infiltration and activation of CD8+ T cells and NK cells within the tumor microenvironment (23, 47). In addition, eosinophils may exert direct cytotoxic effects through the release of granule proteins and further shape a microenvironment more permissive to immune-cell function by modulating tumor vasculature and local inflammatory conditions (23, 48). Eosinophil-derived mediators may also contribute to vascular normalization and promote macrophage polarization toward an M1-like antitumor phenotype (23). Taken together, these mechanisms suggest that an increase in peripheral eosinophils during treatment may serve as a surrogate of enhanced ICI-induced immune activation. Accordingly, dynamic changes in AEC and E% during therapy may represent promising biomarkers for predicting both the efficacy and safety of immunotherapy (49).

Given that baseline eosinophil indices are more susceptible to short-term influences such as acute infections and medications, we placed greater emphasis on eosinophil dynamics after ICI exposure, when a sustained state of immune activation has been established. Uchida T et al. reported in a nivolumab monotherapy cohort that early increases in eosinophils during treatment were associated with better clinical responses and longer PFS and OS (50). Okauchi S et al. found that during ICI monotherapy, a peak E% of ≥5% predicted a longer time to treatment failure (TTF) and was an independent prognostic factor, whereas baseline eosinophils were less informative (51). Consistent with these studies, we found that increases in peripheral blood AEC and E% after the initiation of ICI therapy were closely associated with better survival outcomes. Using the minimum p-value approach, we identified optimal cut-off values for changes from baseline to cycle 4 of 0.01×10^9^/L for AEC and 0.85% for E%; patients with increase above these cutoffs had better OS and PFS. Therefore, in clinical practice, dynamic changes in peripheral AEC may be more informative than a single baseline measurement. Our findings highlight the importance of longitudinal monitoring of eosinophils during immunotherapy and provide a potential reference for the individualized management of advanced NSCLC patients receiving ICIs.

Unlike most previous studies, which primarily focused on ICI monotherapy (40, 41, 49), our study better reflects contemporary clinical practice, in which chemoimmunotherapy has become the predominant treatment approach, and therefore provides a more pragmatic assessment of the predictive value of eosinophil-related indices. Subgroup analyses showed that, in the chemoimmunotherapy group, elevated eosinophil levels were significantly associated with more favorable treatment responses, whereas no comparable association was observed in the ICI monotherapy group. Several factors may account for this difference. First, the monotherapy subgroup was relatively small in our cohort, comprising only 12.7% of the overall sample, which limited statistical power and may have increased the risk of false-negative findings, such that true associations in the monotherapy group may have gone undetected. Second, chemotherapy can induce immunogenic cell death, leading to the release of large amounts of tumor antigens and the activation of a broader and more robust antitumor immune response. In this context, the association between dynamic eosinophil changes and treatment efficacy may have been amplified in the combination-therapy group, making it more readily detectable. In addition, although the multivariable models adjusted for several potential confounders, residual and unmeasured confounding cannot be fully excluded given the retrospective design of the study. Taken together, the differences between the two treatment groups should be interpreted cautiously and require further validation in future studies.

Furthermore, our study revealed that the association between irAEs and eosinophil trends depended on the timing of eosinophil count elevation. Notably, higher AEC levels at cycle 2 were significantly associated with an increased incidence of irAEs, whereas no such association was observed at baseline. We then further analyzed changes in eosinophil-related indices to determine how variations at different intervals influenced the occurrence of irAEs. We found that the absolute increase in AEC from baseline to cycle 2 was significantly associated with irAE development, whereas changes from baseline to cycle 4 and from cycle 2 to cycle 4 did not show statistically significant associations. In addition, we found that a higher AEC at cycle 2, as well as an increase in AEC from baseline to cycle 2, was closely associated with the development of severe irAEs (grade ≥3). These findings underscore the potential of early eosinophil dynamics to predict both the occurrence and severity of irAEs, suggesting that monitoring eosinophil changes during the initial phase of immunotherapy may provide valuable information for the early identification of clinically significant adverse events. Previous studies have also supported this view, Tasaki Y et al. reported that AEC measured before two cycles of anti–PD–1 or combined anti–CTLA–4 plus anti–PD–1 therapy was higher in patients who developed irAEs than in those who did not (52). Similar findings have been reported in other tumor types. In metastatic renal cell carcinoma, although baseline eosinophil levels did not differ between patients with and without severe irAEs (2.8% vs 2.5%; p=0.75), E% at cycle 2 was significantly higher in the severe irAE group (mean 6.6% vs 3.3%; p<0.05) (52). It should be noted that the number of grade ≥3 irAE events in this study was limited, resulting in insufficient statistical power for the corresponding analyses. Therefore, the associations between eosinophil-related indices and severe irAEs (grade ≥3) should be interpreted with caution. Therefore, the relationship between eosinophil-related indices and irAEs warrants further investigation in larger, multicenter, prospective cohort studies to improve the robustness, clinical applicability, and accuracy of the findings.

In this study, patients with a history of surgery or local radiotherapy had a higher incidence of irAEs, suggesting that prior local treatment may influence the host immune milieu. Previous studies have shown that radiotherapy can increase tumor antigen exposure and enhance systemic immune activation, thereby potentially augmenting the immune response to subsequent ICI therapy, while also increasing the risk and severity of irAEs (53–55). Although surgery may induce short-term inflammatory responses and alter immune status (56), the available evidence regarding the association between surgical history and irAEs remains limited and inconsistent. One NSCLC cohort study reported that a history of cancer-related surgery before ICI treatment was not significantly associated with an increased overall risk of irAEs (57). In contrast, a prospective real-world cohort study involving patients with HIV and cancer found that prior cancer surgery was significantly associated with an increased risk of severe irAEs (grade ≥3) (58). However, because the latter study was conducted in a special population with underlying immune deficiency, the generalizability of its findings to the broader population of patients with solid tumors remains uncertain. In our cohort, most patients received ICIs after disease recurrence or progression, with a relatively long interval from their initial surgery; therefore, acute postoperative inflammation was unlikely to be a major contributor to irAE development. The observed association between surgical history and irAEs may instead reflect differences in host immune background, tumor burden, or other underlying disease characteristics, rather than a direct effect of prior surgery itself.

Previous studies have largely focused on eosinophil-related indices at baseline or at a single early treatment timepoint and have typically evaluated only one parameter, either AEC or E%. In contrast, our study incorporated both AEC and E% and systematically assessed the associations of their absolute values and dynamic changes across multiple treatment timepoints (baseline, cycle 2, and cycle 4) with the efficacy and safety of ICIs. This study design overcomes the limitations of relying on a single timepoint or a single eosinophil parameter, allowing for a more comprehensive characterization of the relationship between eosinophil dynamics and immunotherapy outcomes, and further supporting their potential clinical value as dynamic biomarkers.

This study has several limitations. First, although we attempted to adjust for confounding factors in the statistical analysis, it is challenging to fully account for all potential confounders; therefore, residual confounding may still be present. Second, cut-off selection using the minimum p-value method can result in over-optimistic p-values and narrower confidence intervals, so further studies are warranted to validate our proposed cutoffs. Third, only patients with locally advanced or advanced lung cancer were included; thus, the impact of eosinophil-related indices on short- and long-term outcomes in patients receiving immunotherapy at earlier disease stages remains to be clarified. Fourth, PD-L1 is one of the most widely used predictive biomarkers for immunotherapy. However, in this real-world cohort, PD-L1 test results were unavailable for a substantial proportion of patients because of insufficient tissue samples, referral from outside institutions, difficulties in follow-up, and incomplete data collection inherent to the retrospective design. Because most patients in this study received chemoimmunotherapy, for which clinical benefit is known to be less dependent on PD-L1 expression status, some patients declined PD-L1 testing. As a result, we were unable to further investigate the potential association between PD-L1 expression and eosinophil-related indices. Fifth, due to incomplete clinical follow-up and adverse event records in some patients, data on irAEs may be partially missing or underestimated. Finally, stratified analyses by treatment regimen suggested differences between the ICI alone and ICI plus chemotherapy groups; however, these findings should be interpreted cautiously given the small sample size of the ICI alone group (n=40), which may have limited statistical power. Despite these limitations, this study provides preliminary evidence for the predictive and prognostic value of eosinophils in the context of immunotherapy. At the same time, it highlights the need for future investigations to employ more rigorous study designs, larger sample sizes, and more comprehensive documentation of adverse events and biomarker profiles. Such prospective multicenter studies will be essential to accurately elucidate the relationships between eosinophil-related indices, irAEs, and clinical outcomes, thereby providing stronger evidence to guide clinical practice.

## 5 Conclusion

This study indicates that higher AEC and E% at cycle 4, as well as increases from baseline to cycle 4, are associated with longer PFS and OS. Regarding safety, higher AEC at cycles 2 and early increases from baseline to cycle 2, were associated with an increased risk of irAEs. Taken together, peripheral AEC may serve as a simple, low-cost biomarker for prognostic assessment and irAE risk stratification in NSCLC patients receiving ICI therapy, a role that warrants confirmation in prospective studies.

## Data Availability

The raw data supporting the conclusions of this article will be made available by the authors, without undue reservation.
