# Supplementary material for: Predictive value of peripheral blood eosinophil levels and dynamics for efficacy and safety of immune checkpoint inhibitors in non-small cell lung cancer: a real-world study
Source: Front Immunol. 2026 Apr 30;17:1816610. doi: 10.3389/fimmu.2026.1816610 (PMC13171796; doi:10.3389/fimmu.2026.1816610)
Supplement: Supplementary file 1 [file DataSheet1.docx]

**Supplemental Table**

**Supplemental Table 1. Longitudinal changes in eosinophil indices during ICI therapy stratified by treatment regimen**

|  | **ICI + chemotherapy** | | **ICI alone** | |  |
| --- | --- | --- | --- | --- | --- |
|  | **Relative fold change** ^a^ **(95% CI)** | ***P*-Value** | **Relative fold change** ^a^ **(95% CI)** | ***P*-Value** | ***P-*Value for interaction** ^b^ |
| **AEC, ×10⁹/L** |  |  |  |  |  |
| Baseline | 1 (Reference) |  | 1 (Reference) |  |  |
| Cycle 2 | 0.65 (0.56, 0.75) | <0.001 | 1.07 (0.72, 1.57) | 0.750 | 0.019 |
| Cycle 4 | 0.72 (0.61, 0.84) | <0.001 | 0.68 (0.44, 1.04) | 0.072 | 0.788 |
| **E%** |  |  |  |  |  |
| Baseline | 1 (Reference) |  | 1 (Reference) |  |  |
| Cycle 2 | 0.79 (0.68, 0.90) | 0.001 | 0.98 (0.68, 1.43) | 0.930 | 0.268 |
| Cycle 4 | 0.92 (0.79, 1.07) | 0.286 | 0.61 (0.41, 0.91) | 0.016 | 0.060 |

^a^ Relative fold change estimated from linear mixed effect models, adjusted for gender, age, smoking status, body mass index, stage, brain metastasis, and line of initial therapy

^b^ Test for interaction between treatment cycle by ICI regimens

Abbreviations: ICI, Immune checkpoint inhibitors; AEC, absolute eosinophil count

**Supplemental Table 2:** Association of eosinophil count and percentage at baseline, cycle 2, and cycle 4, as well as their changes, with OS and PFS, stratified by treatment regimen.

|  |  | **ICI + chemotherapy** | | **ICI alone** | |  |
| --- | --- | --- | --- | --- | --- | --- |
|  |  | **Adjusted HR** ^a^  **(95 % CI)** | ***P*-Value** | **Adjusted HR** ^a^  **(95 % CI)** | ***P*-Value** | ***P-*Value for interaction** ^c^ |
| **OS** ^b^ | **Absolute Eosinophil Count** |  |  |  |  |  |
|  | Baseline | 0.93 (0.83, 1.05) | 0.258 | 1.06 (0.73, 1.54) | 0.753 | 0.516 |
|  | Cycle 2 | 0.98 (0.87, 1.10) | 0.741 | 0.94 (0.68, 1.29) | 0.688 | 0.797 |
|  | Cycle 4 | 0.79 (0.69, 0.91) | 0.001 | 1.09 (0.80, 1.49) | 0.581 | 0.063 |
|  | Change Baseline to Cycle 2 | 1.02 (0.90, 1.16) | 0.748 | 0.89 (0.65, 1.23) | 0.480 | 0.440 |
|  | Change Baseline to Cycle 4 | 0.87 (0.76, 0.99) | 0.039 | 1.02 (0.79, 1.31) | 0.905 | 0.267 |
|  | Change Cycle 2 to Cycle 4 | 0.85 (0.73, 0.98) | 0.022 | 1.19 (0.86, 1.64) | 0.304 | 0.065 |
|  | **Eosinophil Percent** | | |  |  |  |
|  | Baseline | 0.92 (0.83, 1.03) | 0.149 | 0.90 (0.61, 1.33) | 0.604 | 0.913 |
|  | Cycle 2 | 0.88 (0.78, 0.99) | 0.047 | 1.03 (0.75, 1.40) | 0.868 | 0.385 |
|  | Cycle 4 | 0.77 (0.67, 0.88) | <0.001 | 1.17 (0.85, 1.61) | 0.336 | 0.016 |
|  | Change Baseline to Cycle 2 | 0.91 (0.80, 1.04) | 0.176 | 1.09 (0.77, 1.55) | 0.624 | 0.355 |
|  | Change Baseline to Cycle 4 | 0.79 (0.68, 0.92) | 0.003 | 1.11 (0.85, 1.45) | 0.444 | 0.026 |
|  | Change Cycle 2 to Cycle 4 | 0.85 (0.73, 0.99) | 0.039 | 1.10 (0.86, 1.41) | 0.449 | 0.084 |
| **PFS** ^b^ | **Absolute Eosinophil Count** | | |  |  |  |
|  | Baseline | 0.98 (0.90, 1.08) | 0.708 | 1.05 (0.73, 1.49) | 0.801 | 0.736 |
|  | Cycle 2 | 1.04 (0.95, 1.14) | 0.440 | 0.94 (0.71, 1.25) | 0.671 | 0.521 |
|  | Cycle 4 | 0.91 (0.82, 1.00) | 0.052 | 0.94 (0.69, 1.27) | 0.672 | 0.844 |
|  | Change Baseline to Cycle 2 | 1.04 (0.94, 1.14) | 0.459 | 0.84 (0.63, 1.10) | 0.217 | 0.163 |
|  | Change Baseline to Cycle 4 | 0.94 (0.86, 1.03) | 0.192 | 0.90 (0.71, 1.14) | 0.389 | 0.745 |
|  | Change Cycle 2 to Cycle 4 | 0.90 (0.82, 0.99) | 0.037 | 1.00 (0.72, 1.39) | 0.988 | 0.561 |
|  | **Eosinophil Percent** | | |  |  |  |
|  | Baseline | 0.97 (0.89, 1.05) | 0.429 | 1.06 (0.79, 1.44) | 0.682 | 0.543 |
|  | Cycle 2 | 0.95 (0.87, 1.05) | 0.303 | 0.99 (0.75, 1.31) | 0.939 | 0.799 |
|  | Cycle 4 | 0.87 (0.79, 0.96) | 0.006 | 0.98 (0.71, 1.35) | 0.895 | 0.506 |
|  | Change Baseline to Cycle 2 | 0.95 (0.86, 1.05) | 0.348 | 0.88 (0.65, 1.20) | 0.429 | 0.650 |
|  | Change Baseline to Cycle 4 | 0.91 (0.82, 0.99) | 0.047 | 0.96 (0.74, 1.24) | 0.731 | 0.700 |
|  | Change Cycle 2 to Cycle 4 | 0.91 (0.82, 1.01) | 0.089 | 1.05 (0.80, 1.37) | 0.744 | 0.362 |

^a^ Adjusted hazard ratio per doubling of eosinophil indices in Cox proportional hazard models, adjusted for gender, age, smoking, body mass index, stage, brain metastasis, and line of initial therapy

^b^ For cycle 2 and cycle 4 analyses, landmark times were set at 42 and 84 days after initiation of ICI therapy, respectively

^c^ Test for interaction between eosinophil index and ICI regimens

Abbreviations: HR, hazard ratio; CI, confidence interval; OS, overall survival; PFS, progression-free survival; ICI, Immune checkpoint inhibitors

Note: Eosinophil indices modeled per doubling

**Supplemental Table 3:** Geometric means of eosinophil indices stratified by irAE grade

|  | **No irAE**  **(n=194)** | **irAE Grade <3**  **(n=97)** | **irAE Grade ≥3**  **(n=24)** | ***P*-Value**^a^ |
| --- | --- | --- | --- | --- |
|  | **Geometric Mean**  **(95% CI)** | **Geometric Mean**  **(95% CI)** | **Geometric Mean**  **(95% CI)** |  |
| **AEC, ×10⁹/L** |  |  |  |  |
| Baseline | 0.09 (0.08, 0.10) | 0.10 (0.08, 0.12) | 0.13 (0.08, 0.19) | 0.128 |
| Cycle 2 | 0.05 (0.04, 0.06) | 0.08 (0.07, 0.10) | 0.21 (0.13, 0.34) | <0.001 |
| Cycle 4 | 0.06 (0.05, 0.07) | 0.09 (0.07, 0.11) | 0.09 (0.04, 0.20) | 0.021 |
| **E%** |  |  |  |  |
| Baseline | 1.19 (1.02, 1.38) | 1.41 (1.14, 1.74) | 1.59 (1.00, 2.51) | 0.098 |
| Cycle 2 | 1.01 (0.86, 1.18) | 1.04 (0.83, 1.32) | 2.49 (1.35, 4.58) | 0.069 |
| Cycle 4 | 1.24 (1.02, 1.51) | 1.18 (0.91, 1.52) | 1.10 (0.50, 2.41) | 0.671 |

^a^*P*-value determined by ordinal logistic regression
